# Supplementary material for: Shape familiarity modulates preference for curvature in drawings of common-use objects
Source: PeerJ. 2021 Jul 6;9:e11772. doi: 10.7717/peerj.11772 (PMC8269663; doi:10.7717/peerj.11772)
Supplement: Supplemental Information 1 — ∗p < .05, ∗∗p < .01, ∗∗∗p <.001. [file peerj-09-11772-s001.docx]

| *Predictor* | *β* | *SE* | *df* | *t* | *p* | *95 % CI* |
| --- | --- | --- | --- | --- | --- | --- |
| Contour | 4.85 | 1.32 | 53 | 3.67 | < .001 *** | -7.4, -2.3 |
| Art interest | .27 | .23 | 26.1 | 1.15 | .26 | -.19, .72 |
| Art knowledge | .33 | .79 | 32.5 | .41 | .68 | -1.2, 1.9 |
| Openness to experience | -.06 | .44 | 39.3 | -.13 | .89 | -.90, .83 |
| Unconventionality | -.44 | .64 | 28.5 | -.70 | .50 | -1.7, .81 |
| Holistic Big Picture | 1.3 | .52 | 23 | 2.5 | .02 * | .28, 2.3 |
| Holistic Abstract | .07 | .83 | 39.8 | .09 | .93 | -.89, 1.5 |
| Affective | .035 | .33 | 36.2 | .10 | .92 | -.63, .52 |
| Inferential | .53 | .42 | 42.28 | 1.24 | .22 | -.13, 1.2 |
| Contour x Art interest | -.006 | .18 | 39.5 | -.033 | .97 | -.37, .36 |
| Contour x Art knowledge | .73 | .59 | 40.1 | 1.23 | .22 | -.45, 1.84 |
| Contour x Openness to experience | .38 | .29 | 39.3 | 1.28 | .21 | -.21, .92 |
| Contour x Unconventionality | -.24 | .44 | 40 | -.56 | .58 | -1.1, .62 |
| Contour x Holistic Big Picture | .52 | .42 | 42.3 | 1.24 | .22 | -.30, 1.35 |
| Contour x Holistic Abstract | -.19 | .53 | 44.5 | -.35 | .73 | -1.2, .85 |
| Contour x Affective | -.12 | .23 | 39.2 | -.53 | .60 | -.58, .33 |
| Contour x Inferential | -.26 | .29 | 42.5 | -.88 | .39 | -.83, .32 |
