# Supplementary material for: Shape familiarity modulates preference for curvature in drawings of common-use objects
Source: PeerJ. 2021 Jul 6;9:e11772. doi: 10.7717/peerj.11772 (PMC8269663; doi:10.7717/peerj.11772)
Supplement: Supplemental Information 2 — ∗p < .05, ∗∗p < .01, ∗∗∗p < .001. [file peerj-09-11772-s002.docx]

| *Predictor* | *β* | *SE* | *Z* | *p* | *95 % CI* |
| --- | --- | --- | --- | --- | --- |
| Art interest | .026 | .017 | 1.52 | .13 | -.007, .06 |
| Art knowledge | -.004 | .045 | -.10 | .92 | -.09, .08 |
| Openness to experience | .006 | .023 | .26 | .80 | -.04, .05 |
| Unconventionality | -.090 | .041 | -2.2 | .028 * | -.17, -.01 |
| Holistic Big Picture | .13 | .037 | 3.5 | < .001 *** | .06, .20 |
| Holistic Abstract | -.006 | .051 | -.12 | .91 | -.11, .09 |
| Affective | .052 | .016 | 3.33 | < . 001 *** | .02, .08 |
| Inferential | .010 | .028 | .37 | .71 | -.04, .06 |
